# Cytomegalovirus-Associated Venous and Arterial Thrombotic Disease

**DOI:** 10.7759/cureus.12161

**Published:** 2020-12-18

**Authors:** Amar H Kelkar, Brian L Loc, Michael D Tarantino, Anita Rajasekhar, Huaping Wang, Mona Kelkar, John Farrell

**Affiliations:** 1 Division of Hematology & Oncology, University of Florida College of Medicine, Gainesville, USA; 2 Department of Cardiology, OSF Saint Francis Medical Center, Peoria, USA; 3 Department of Hematology, Bleeding & Clotting Disorders Institute, Peoria, USA; 4 Department of Medicine, University of Illinois College of Medicine at Peoria, Peoria, USA; 5 Department of Biostatistics & Epidemiology, Harvard School of Public Health, Cambridge, USA; 6 Department of Microbiology & Immunology, OSF System Laboratory, Peoria, USA

**Keywords:** acquired coagulation disorders, venous thrombosis, arterial thrombosis, viral infection, cytomegalovirus, cmv, venous thromboembolism, viral host interactions, acute coronary syndromes, thrombo embolic disease

## Abstract

Background: Cytomegalovirus (CMV) infection has been associated with venous thromboembolism (VTE) and acute coronary syndromes (ACS).

Methods: A retrospective study was conducted within the OSF HealthCare System in Peoria, IL. The objectives were to determine the incidence of acute VTE and ACS within one year of CMV testing. The “study group” included patients with positive CMV immunoglobulin M (IgM) or positive CMV polymerase chain reaction (PCR). The “seropositive control” group included patients with positive CMV immunoglobulin G (IgG) and negative IgM. The “seronegative control” group included patients with negative CMV IgG and IgM, or negative PCR.

Results: Within one year of CMV infection, 38 of 379 patients (10.0%) developed VTE in the study group compared to 41 of 1334 patients (3.1%) in the seropositive control and 37 of 1249 (3.0%) in the seronegative control. Adjusting for age and gender, both control groups were less likely to have VTE than the study group within one year (seropositive control: odds ratio (OR) = 0.3, 95% confidence interval (CI) 0.2-0.5, p < 0.0001; seronegative control: OR = 0.4, 95% CI 0.2-0.6, p < 0.0001). ACS was more likely to occur in the study group, with the incidence of 7.7% compared to 4.7% (p < 0.0001) in the seropositive control and 1.9% (p <0.0001) in the seronegative control. Adjusting for age and gender, the seronegative control was less likely to develop ACS than the study group within one year (OR = 0.4, 95% CI 0.2-0.7, p = 0.003).

Conclusions: This retrospective study demonstrates that CMV infection may be a significant risk factor for VTE and ACS.

## Introduction

Cytomegalovirus (CMV) is a heterogeneous DNA virus in the herpesviridae family capable of infecting a broad range of tissue types within a host [1]. This virus has been associated with a wide variety of chronic diseases [2]. CMV infection is rarely associated with disease in immunocompetent individuals, although it may cause heterophile-negative mononucleosis. CMV is medically important and causes invasive infections in primary immunocompromised hosts, patients on immunosuppressants, and in newborns following transplacental infection during pregnancy [1].

Venous thromboembolism (VTE), including deep vein thrombosis (DVT), pulmonary embolism (PE), portal vein thrombosis (PVT), and other splanchnic vein thromboses may be attributed to reversible and irreversible provoking risk factors. Numerous risk factors have been linked to VTE. Irreversible risk factors include inherited thrombophilic disorders, advanced age, prior VTE, and underlying malignancy. Reversible risk factors include recent surgery, trauma, prolonged immobility, obesity, pregnancy, estrogen use, indwelling catheters, acute infection, and current or recent hospitalization [3,4]. The VTE risk associated with each of these is dependent on the context and varies throughout published literature [3,5]. However, few studies have explored CMV as a risk factor for VTE [6]. Current guidelines recommend transient or reversible causes of VTE be treated with anticoagulation for three months, whereas unprovoked VTE is treated with extended anticoagulation unless the bleeding risk is high [7]. CMV was first described in association with venous thromboembolism in 1984. Since that time, dozens of cases of CMV-associated VTE in both immunocompromised and immunocompetent hosts have been described [8].

Acute coronary syndrome (ACS) is a conglomeration of disease states including ST-elevation myocardial infarction (STEMI), non-ST elevation myocardial infarction (NSTEMI), and others delineated in recent consensus guidelines [9,10]. The majority of ACS is the result of lipid-rich plaque rupture promoting a localized prothrombotic state leading to arterial thrombosis and vascular occlusion. Standard ACS therapy includes a combination of antiplatelet agents, anticoagulants, and mechanical interventions. Factors promoting prothrombotic states have been shown to increase the incidence of ACS [9,10]. CMV has also been associated with acute coronary syndrome and other arterial vascular diseases. CMV antigens were first identified in cultured human arterial smooth muscle cells from atherosclerotic carotid and aortic plaques and this relationship has been further explored in vitro; however, results in vivo have been limited [11,12].

Herein, we present retrospective data collected from the OSF HealthCare System to determine the incidence of acute VTE and ACS within one year of CMV testing. The purpose was to determine whether biological models of CMV thrombogenicity translate to increased incidence of thrombotic events.

## Materials and methods

We conducted an institutional retrospective study of patients with prior CMV testing within the OSF HealthCare System, a network of 13 hospitals based in Peoria, IL, between January 2008 and September 2017. During this period, 2,144,725 patients were seen across these facilities. This study was performed by a query of the Epic Systems™ (Verona, WI) patient electronic health record. Patients with CMV immunoglobulin G (IgG), immunoglobulin M (IgM), or polymerase chain reaction (PCR) testing, or an ICD-9 (078.5) or ICD-10 (B25.9) diagnosis code for CMV disease during the study period were included. CMV immunoglobulin testing was performed in the OSF System Laboratory on a BioPlex® 2200 automated analyzer (Bio-Rad Laboratories, Hercules, CA). CMV deoxyribonucleic acid (DNA) polymerase chain reaction (PCR) is a plasma-based assay performed in the OSF System Laboratory with the Cobas® AmpliPrep/Cobas® TaqMan® CMV quantitative PCR (Roche Diagnostics, Indianapolis, IN). A data collection form was developed for systematic retrieval of anonymized epidemiologic, historic, diagnostic, and treatment data. Patients of all ages including children and pregnant women were included, but incarcerated patients were excluded. Data were initially collected by database query and then subsequently verified and completed by manual chart review by two of the study investigators. The study was approved by the OSF HealthCare System Research Administration Department and the University of Illinois College of Medicine at Peoria Institutional Review Board.

The primary outcome was the incidence of acute VTE within one year of CMV testing. These data were collected by database query of the sample population within one year of the CMV detection date, initially using ICD-9 (415.1, 452, 453.4, 453.8) and ICD-10 (I26.90, I26.99, I81, I82, I82.40, I82.90) codes for VTE and all subtypes to identify diagnosis and date. Manual review of the charts was performed to confirm diagnoses using available imaging or provider documentation. VTE was defined as deep vein thrombosis of the upper or lower extremities, pulmonary embolism, or splanchnic vein thromboses. Catheter-related DVTs were included. PE was confirmed by results of computed tomography with pulmonary angiography (CTPA). DVT or splanchnic vein thrombosis was confirmed by venous doppler ultrasound, abdominal ultrasound, or computed tomography with angiography. The majority of events were confirmed by in-network studies; however, scanned reports in the electronic health record from outside facilities were also reviewed and incorporated. Demographic data were also collected.

Secondary outcome data included events of ACS within one year of CMV testing. These data were also collected by database query of the sample population within one year of the CMV detection date, initially using ICD-9 (410) and ICD-10 (I21) codes to identify diagnosis and date, followed by manual review of the charts to confirm these diagnoses using available imaging, provider documentation, or scanned reports from outside facilities. ACS was defined as ST-elevation myocardial infarction and non-ST elevation myocardial infarction, confirmed by coronary angiography or a combination of elevated troponin levels with consistent electrocardiogram findings for NSTEMI [9,10].

Data were de-identified after extraction and review. Univariate and multivariate analysis included logistic regression models, chi-square test, Kruskal-Wallis test, and Wilcoxon two-sample test, followed by the post hoc test once statistical significance was determined, all performed using SAS® 9.4 software (Cary, NC). Age- and gender-adjusted analysis was also performed. Patient race was collected with initial demographic data but was not consistently recorded and was thus excluded from further analysis.

## Results

**Figure 1:**
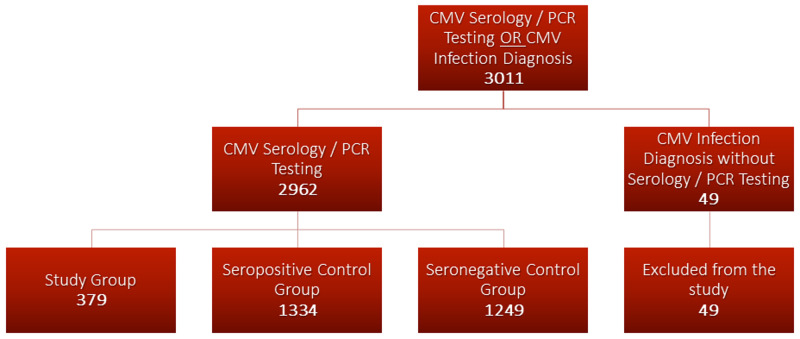
Study Design. The patient population was divided into three groups after excluding patients with no serology or PCR testing: “study group” (IgM+/PCR+), “seropositive control” group (IgG+/IgM-/PCR-), “seronegative control” group (IgG-/IgM-/PCR-). PCR: polymerase chain reaction; CMV: cytomegalovirus; IgG: immunoglobulin G; IgM: immunoglobulin M

The initial sample consisted of 3011 patients, and the medical records were manually reviewed for quality-control. Forty-nine patients were excluded due to lack of CMV IgG, IgM, or DNA PCR testing to confirm the date of CMV diagnosis. The remaining population of 2962 patients was then divided into three groups. The “study group” included all patients with acute or recent CMV infection, defined as positive CMV IgM or detection of CMV DNA by PCR in plasma. The remaining patients were divided into two control groups. The “seropositive control” group was defined as patients with positive CMV IgG and negative IgM or PCR. The “seronegative control” group was defined as patients with negative CMV IgG, IgM, and/or PCR. Three patients included in the study group had positive CMV PCR, but negative IgM testing. The study design is depicted in Figure 1.

The study population of patients with acute CMV infection consisted of 379 patients. The seropositive control group included 1334 patients. The seronegative control group included 1249 patients.

Statistical differences between the groups were identified by the post hoc test. Patients in the study group (median 38.4 years, interquartile range (IQR) 39.2) were older than both the seropositive control group (median 28.4 years, IQR 43.1, p < 0.0001) and the seronegative control group (median 18.7 years, IQR 32.9, p < 0.0001). The seropositive control group was older than the seronegative control group (p < 0.0001). The seropositive control group also had fewer male patients than the study group (41.8% versus 51.2%, p = 0.001) and the seronegative control group (41.8% versus 48.9%, p = 0.0003). Demographic data of age and gender can be seen in Table 1. The median age of the patients with VTE in the study group was 60.6 years (IQR 22.1), compared to 50.47 years (IQR 48.0) in the seropositive control group and 37.6 years (IQR 44.4) in the seronegative control group.

**Table 1:** Demographics. Description of age and gender demographics within the seropositive control group, seronegative control group, and study group. IQR: interquartile range

Demographics				
		Seropositive Control Group	Seronegative Control Group	Study Group
		n = 1334	n = 1249	n = 379
Age, years				
	Median (IQR)	28.4 (43.1)	18.7 (32.9)	38.4 (39.2)
Gender, n (%)				
	Female	776 (58.2)	638 (51.1)	185 (48.8)
	Male	558 (41.8)	611 (48.9)	194 (51.2)

**Figure 2:**
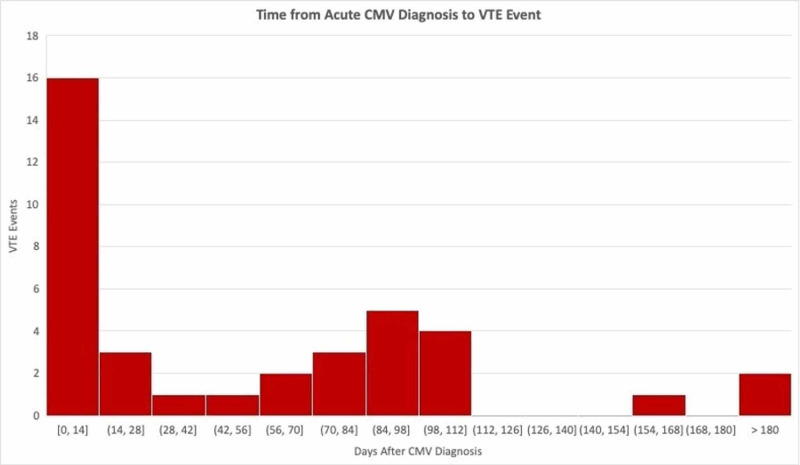
Time From Acute CMV Diagnosis to VTE Event. Within the study group, the majority of VTE events occurred within six months of the initial acute CMV diagnosis, supporting a temporal relationship. VTE: venous thromboembolism; CMV: cytomegalovirus

For primary outcome data, 38 of 379 patients (10.0%) in the study group were diagnosed with VTE within one year of acute CMV diagnosis. The vast majority of these events occurred within the first six months, as depicted in Figure 2. This is compared to 41 of 1334 patients (3.1%) in the seropositive control group and 37 of 1249 patients (3.0%) in the seronegative control group (Table 2). The seropositive control group had a lower incidence of VTE than the study group (3.1% versus 10.0%, p < 0.0001). The seronegative control group also had a lower incidence of VTE than the study group (3.0% versus 10.0%, p < 0.0001). There was no statistically significant difference between the incidence of VTE in the seropositive control group and seronegative control group (3.1% versus 3.0%, p = 0.9), suggesting that the effect of acute CMV infection on VTE incidence may be transient.

**Table 2:** Incidence of VTE and ACS in Control Groups Versus Study Group. Incidence of VTE and ACS in the seropositive control group, seronegative control group, and study group with calculated OR, 95% CI, and p values between each of the control groups and the study group. Age- and gender-adjusted results were also calculated. VTE: venous thromboembolism; ACS: acute coronary syndromes; OR: odds ratio; CI: confidence interval

	Seropositive Control Group	Seronegative Control Group	Study Group		Unadjusted Results		Age- and Gender- Adjusted Results	
	n = 1334	n = 1249	n = 379		p value	OR (95% CI)	p value	OR (95% CI)
VTE, n (%)	41 (3.1)	37 (3.0)	38 (10.0)	Seropositive Control Group vs. Study Group	<0.0001	0.3 (0.2-0.5)	<0.0001	0.3 (0.2-0.5)
				Seronegative Control Group vs. Study Group	<0.0001	0.3 (0.2-0.4)	<0.0001	0.4 (0.2-0.6)
				Seropositive Control Group vs. Seronegative Control Group	0.9	1.0 (0.7-1.6)	0.5	0.9 (0.5-1.4)
ACS, n (%)	62 (4.7)	24 (1.9)	29 (7.7)	Seropositive Control Group vs. Study Group	0.02	0.6 (0.4-0.9)	0.2	0.7 (0.4-1.2)
				Seronegative Control Group vs. Study Group	<0.0001	0.2 (0.1-0.4)	0.003	0.4 (0.2-0.7)
				Seropositive Control Group vs. Seronegative Control Group	0.0002	2.5 (1.5-4.0)	0.03	1.8 (1.1-2.9)

Multivariate analysis was performed after adjustment for age and gender. VTE in the study group was higher than either the control group (Table 2). The seropositive control group was less likely to have VTE than the study group within one year (OR = 0.3, 95% confidence interval (CI) 0.2-0.5, p < 0.0001). The seronegative control group was also less likely to have VTE than the study group within one year (OR = 0.4, 95% CI 0.2-0.6, p < 0.0001).

Secondary outcome data demonstrated that ACS was more likely to occur in the study group, with an incidence at one year of 7.7%, compared to 4.7% (p < 0.0001) in the seropositive control group and 1.9% (p < 0.0001) in the seronegative control group (Table 2). Multivariate analysis of incidence adjusted for age and gender demonstrated that the seronegative control group was less likely to develop ACS than the study group within one year (OR = 0.4, 95% CI 0.2-0.7, p = 0.003). However, the seropositive control group failed to demonstrate significant differences compared the study group (OR = 0.7, 95% CI 0.4-1.2, p = 0.2). The difference between the two control groups in the secondary outcomes data was significant, with the seropositive control group more likely to develop ACS within one year than the seronegative control group (OR = 1.8, 95% CI 1.1-2.9, p = 0.03).

## Discussion

This study sought to retrospectively determine the incidence of acute VTE in the one-year period following CMV testing, dividing the results into groups of patients with an active infection, seropositive IgG testing without active infection, and seronegative IgG and IgM testing. Differences in the incidence of acute VTE between these groups suggest that CMV may have contributed to a prothrombotic state in patients with active infection or seropositive state.

These data align with several studies in the past decade supporting the association between CMV and VTE. The MAISTHRO retrospective registry case-control study reported 4.3% with CMV IgM positivity amongst patients with acute VTE, compared to 0.6% with CMV IgM positivity in matched control patients without acute VTE (OR = 7.3) [13]. One large single-center prospective case-control study observed patients with acute CMV infection and matched CMV IgM negative controls for 6 months and found 3.06 cases of acute VTE per 1000 in those with positive IgM compared to 1.36 cases of acute VTE per 1000 in those with negative IgM (OR = 2.25, 95% CI 1.38-3.66, p = 0.003) [6]. A retrospective case-control study of 140 patients with acute CMV infection showed an incidence of concurrent VTE of 2.9%, which was significantly greater than 0% VTE in 140 matched controls without acute CMV infection [14]. A meta-analysis concluded that between 1.9% and 9.1% of patients hospitalized with acute venous thromboembolism had concurrent acute CMV infection [15].

Our study is the second to look at the incidence of acute VTE following acute CMV infection over a prolonged period of time compared to the incidence of acute VTE without acute CMV infection, following the work of Paran and colleagues [6]. We chose one-year follow-up, compared to six-month follow-up in prior studies, to increase the likelihood of capturing potential CMV-related VTE and ACS events based on existing data suggesting a durable effect of increased thrombosis, as well as the chronic nature of the viral infection. A unique feature was that our study demonstrated differences between acute CMV infection and seropositive status without active infection. The incidence of acute VTE in the seropositive control group was not significantly different than the incidence in the seronegative control group. The data provide additional evidence for CMV being a reversible or transient risk factor of VTE. Current guidelines recommend transient or reversible causes of VTE be treated with anticoagulation for 3 months, whereas unprovoked VTE is treated with extended anticoagulation unless the bleeding risk is high [7]. We suspect that CMV-associated VTE is likely underrecognized because CMV testing is not routinely performed outside of immunocompromised populations [8]. However, additional retrospective and prospective studies are needed to determine whether acute CMV infection is a reversible risk factor for acute VTE and can be used to change CMV screening, primary prophylaxis, and VTE treatment practices.

The secondary outcome data collected in this study similarly showed a measurable difference between the seronegative control group and the study group, regarding the incidence of ACS. However, the incidence of ACS was not significantly different between the seropositive control group and the study group. While there are no prior studies of similar design to directly compare these data, the discrepancies might be explained by the different mechanisms involved in arterial thrombosis. Studies examining the role of CMV in arterial disease fall into two groups. CMV IgG seropositivity and chronic CMV endotheliitis have been linked with the pathogenesis of atherosclerosis, while acute CMV infection has been associated with ACS [11,12,16-18]. Both primary ACS and restenosis were counted amongst the ACS incidence data in our study, possibly explaining the similar incidence rate of ACS in both the seropositive control group and the study group.

Our study data should be considered in the context of the following limitations to the study design. Data were collected retrospectively and without prejudice towards the immunocompromised status or reason for CMV testing, though we suspect that most of the patients were immunocompromised or acutely ill to justify the CMV testing. There was a large variance in the justification for CMV testing across the spectrum of patients in the initial study population and clinical symptoms of infection were not consistently recorded. Clinical history along with both immunoglobulin and serum DNA testing was not always available, and in a few cases, CMV IgM was used as a surrogate marker, limiting differentiation between acute, chronic, recurrent, and recent CMV infections. When designing the study, we noted three patients in the study group that had positive CMV PCR, but negative IgM testing. These patients were included in the study group based on the study design and due to high serum PCR titers. One limitation of the CMV PCR studies was that in some cases, only qualitative studies were available, limiting analysis on the correlation between CMV DNA titers and acute VTE. The method of database query could not account for VTE or ACS events occurring outside of the OSF HealthCare System, conferring a form of attrition bias, though scanned records sent from out-of-system facilities were reviewed and included in the analysis. There was an unusually high rate of VTE incidence across all the groups within this study, likely due to other concurrent risk factors for VTE. However, other risk factors for VTE and CMV infection, including immunocompromised status, were not collected in the data set and thus could not be used for adjustment in a multivariate analysis. We also did not collect data on other viral infections to account for coinfections or for comparison in VTE and ACS incidence. The data did not differentiate between primary ACS and restenosis, due to limitations in the database query. Furthermore, treatment with antiviral medications was not captured in the data and thus its impact could not be included in the analysis. These study limitations are also described in Table 3.

**Table 3:** Study Limitations. The results of the study must be viewed within the context of its limitations, which include its retrospective study design, large volume of patients with missing or untested data points, and database search limitations. CMV: cytomegalovirus; VTE: venous thromboembolism; DNA: deoxyribonucleic acid; ACS: acute coronary syndromes

Study Limitations
Design Limitations
Retrospective study design; data were collected without prejudice towards the immunocompromised status or reason for CMV testing; did not collect data on concurrent risk factors of VTE or immunocompromised status for multivariate analysis
Database Limitations
Unusually, high rate of VTE incidence across all the groups within this study; clinical symptoms of infection were not consistently recorded; large variance in the justification for CMV testing; both immunoglobulin and serum DNA testing were not always available, limiting differentiation between acute, chronic, recurrent, and recent CMV infections; CMV DNA PCRs were sometimes ordered only as quantitative studies, limiting analysis based on levels of CMV viral titers; treatment with antiviral medications not consistently recorded; could not account for VTE or ACS events occurring outside of the OSF HealthCare System; attrition bias; scanned records sent from out-of-system facilities were reviewed and included in the analysis; could not differentiate between primary ACS and restenosis

Despite these limitations, these data support the existence of CMV-associated VTE. There are also several known and proposed mechanisms for a causative relationship that have been described in vitro. Many models are based on host monocyte and endothelial cell infection. Direct infection of vascular endothelial cells has been demonstrated to increase the release of von Willebrand factor and increase cell-surface expression of tissue factor [12,19]. This has been shown to promote inhibition and depletion of natural anticoagulants as well as inhibition of antithrombin III and fibrinolysis [12,20]. CMV infection also increases leukocyte adhesion to CMV-infected endothelial cells, which has been shown to promote a procoagulant microenvironment through similar mechanisms, possibly acting as cofactors [21,22]. Direct infection of monocytes also resulted in increased secretion of tissue factor, thus promoting thrombosis [12]. Another model described an immunologic response to CMV envelope phospholipids, including phosphatidylserine, which has procoagulant prothrombinase properties, reducing the factor Xa clotting time by bypassing most of the coagulation cascade and directly activating thrombin [20].

One proposed model describes transient elevations of antiphospholipid antibodies in the context of acute CMV and other viral infections, including Epstein-Barr Virus, hepatitis C virus, parvovirus, varicella-zoster virus, and adenovirus [23]. A study in mice demonstrated that immunization with a CMV capsule peptide known as TIFI (an analog of human beta-2-glycoprotein I [β2GPI]), induced lupus anticoagulant activity and resulted in thrombotic complications [24,25]. A subsequent study demonstrated that co-injection of TIFI with exogenous antiphospholipid antibodies decreased the incidence of vascular thrombosis in the setting of mechanical injury to the mice. These mice did not form endogenous antiphospholipid antibodies, suggesting a potentially protective effect of the exogenous antibodies [26]. Case reports in humans have described transient increases of serum antiphospholipid antibodies in acute CMV, specifically anticardiolipin IgM [27,28]. This would support the applicability of the findings in mice to humans. However, at this time there are no published studies connecting TIFI to the formation of anti-β2GPI antibodies in human patients [29]. If this link can be established, the homology between CMV TIFI and the 5th domain of human β2GPI could explain why CMV has been more commonly associated with VTE than other viral infections.

Several prior studies have looked at associations between organ system-specific acute infections and thrombosis in the hospital and community settings. One study through the UK-based Health Improvement Network looked at a cohort of patients with first-time diagnoses of DVT (n = 7278) or PE (n = 3755) and looked at the incidence of preceding urinary tract and respiratory tract infections in the preceding six months. They found that the incidence risk ratios of DVT (2.10, 95% CI 1.56-2.82) and PE (2.11, 95% CI 1.38-3.23) were significantly higher after urinary tract infections, particularly in the first two weeks after infection. Respiratory tract infections also had an increased risk of VTE, but diagnostic data were more confounded [30]. One key difference in our study design was following a single infection across a broad cohort of patients, rather than looking for the incidence of preceding infection in a cohort of patients with VTE. Additionally, past studies did not consider the disease-specific causes of VTE. They cite studies discussing transient alterations to endothelial function, increased inflammation measured by surrogate markers such as C-reactive protein, the role of neutrophil activation and neutrophil extracellular traps, tissue factor activation of thrombotic pathways, inhibition of endogenous anticoagulant pathways, and inhibition of fibrinolysis [4,30]. While there are other known associations between acute infection and thrombosis, they neither address nor negate the evidence presented for a potentially unique causal relationship between CMV and thrombosis. This is further evidenced by the presence of increased odds of VTE in the CMV seropositive group of patients suggesting a more prolonged prothrombotic state than has been presented in other infection models of thrombosis.

Additional in vivo studies are needed to further clarify the relationship between acute CMV infection and thrombosis and account for other known risk factors for VTE, especially in high-risk populations. These studies should include data on the incidence of anti-β2GPI antibody formation based on in vitro findings. Future studies must consider the immunocompromised status of patients and the categorization of patients with acute versus chronic recurrent CMV states seen in immunocompromised patients. Future studies could also consider coinfections or comparison to VTE rates of other similar viruses such as Epstein-Barr Virus, Herpesviruses, or other prothrombotic viruses such as severe acute respiratory syndrome coronavirus 2 (SARS-CoV2). Additionally, no prior studies have evaluated VTE prophylaxis in patients with acute or chronic CMV infections, particularly in immunocompromised patients at high risk for CMV recurrence. These concepts should be considered in future clinical trials if an association between CMV infection and VTE is confirmed (Table 4).

**Table 4:** Future Directions. There are many future directions for potential study to better understand the proposed relationship between CMV infection and thrombosis based on the findings of this study. VTE: venous thromboembolism; CMV: cytomegalovirus; PCR: polymerase chain reaction

Future Directions
Studies
Comparative studies in targeted populations such as patients on immune suppression or with active hematologic malignancies; comparative studies to other viruses or evaluation for possible coinfections; clinical trials of VTE prophylaxis in acute CMV infections
Data Collection and Surrogate Markers
Concurrent risk factors of VTE; immunocompromised status; other medical history; anti-β2GPI antibody levels and other antiphospholipid antibody levels at time of acute VTE; quantitative CMV PCR levels; baseline VTE occurrence rate within the same population and setting; CMV infectious symptoms; reasoning for CMV testing; immune suppression medications; use of antiviral CMV medications

## Conclusions

Our findings support the hypothesis of a link between acute CMV infection and thrombotic events such as acute VTE and ACS. In particular, the adjusted odds ratios for acute VTE were convincing of a difference between the study and control populations related to CMV status. The secondary outcome data contribute to the limited existing evidence for the role of acute CMV infection in ACS.
